# On the Centennial of Vitamin D—Vitamin D, Inflammation, and Autoimmune Thyroiditis: A Web of Links and Implications

**DOI:** 10.3390/nu14235032

**Published:** 2022-11-26

**Authors:** Leonidas H. Duntas, Krystallenia I. Alexandraki

**Affiliations:** 1 Thyroid Section, Unit of Endocrinology, Diabetes and Metabolism, Evgenideion Hospital, National and Kapodistrian University of Athens, PC 11528 Athens, Greece; 2 2nd Department of Surgery, Aretaieio Hospital, National and Kapodistrian University of Athens, PC 11528 Athens, Greece

**Keywords:** vitamin D, autoimmune thyroiditis, antithyroid peroxidase antibodies, antithyroglobulin antibodies, 1-α-hydroxylase, TSH

## Abstract

The 100th anniversary of the discovery of vitamin D3 (VitD3) coincides with significant recent advances in understanding its mechanism of action along with accumulating knowledge concerning its genomic and nongenomic activities. A close relationship between VitD3 and the immune system, including both types of immunity, innate and adaptive, has been newly identified, while low levels of VitD3 have been implicated in the development of autoimmune thyroiditis (AIT). Active 1,25(OH)_2_ D3 is generated in immune cells via 1-α-hydroxylase, subsequently interacting with the VitD3 receptor to promote transcriptional and epigenomic responses in the same or adjacent cells. Despite considerable progress in deciphering the role of VitD3 in autoimmunity, its exact pathogenetic involvement remains to be elucidated. Finally, in the era of coronavirus disease 2019 (COVID-19), brief mention is made of the possible links between VitD3 deficiency and risks for severe COVID-19 disease. This review aims to commemorate the centennial of the discovery of VitD3 by updating our understanding of this important nutrient and by drawing up a framework of guidance for VitD3 supplementation, while emphasizing the necessity for personalized treatment in patients with autoimmune thyroid disease. A tailored approach based on the specific mechanisms underlying VitD3 deficiency in different diseases is recommended.

## 1. Introduction

**Figure 1:**
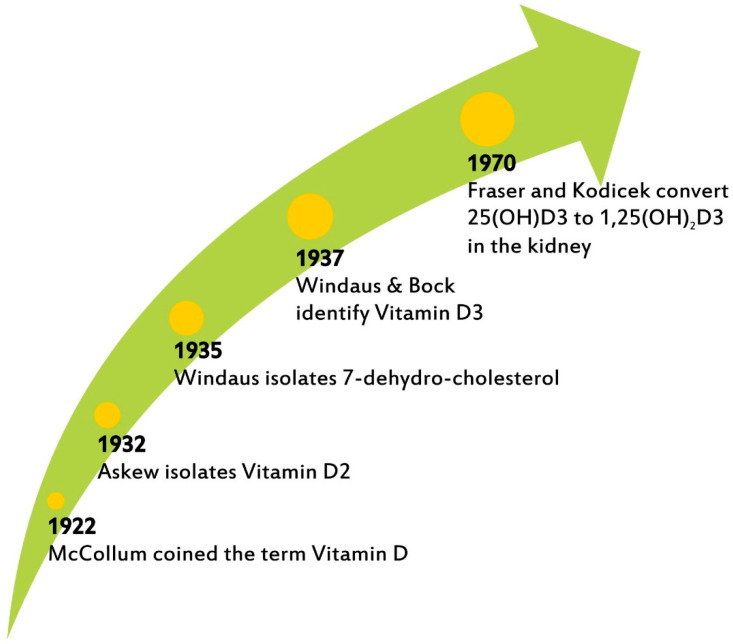
The chronography of VitD3 discovery.

The term vitamin D (VitD) was coined in 1922 by McCollum, who recognized its importance for the promotion of bone growth and the prevention of rickets [1]. Askew elucidated its structure a decade later; Windaus and Bock identified vitamin D3 in 1937; Fraser and Kodicek described the biosynthesis of active VitD3 by the kidney in 1970 [2,3,4] (Figure 1). This year marks the centennial of the naming of VitD3, a micronutrient about which there is increasing interest today not only in its well-established therapeutic action in rickets and osteoporosis, as the main regulator of calcium and phosphorous but also, most notably, in its impact on inflammation and cardiovascular risk as well as its effects on the endocrine and immune systems [5].

Remarkable new findings regarding the non-genomic effects of VitD3 on various tissues underline the need for adequate VitD3 levels for the prevention of an array of diseases and for the maintenance of health [6,7]. Crucially, its anti-inflammatory, antioxidant, and neuroprotective properties also support immune and muscle function and brain cell activity [8,9].

However, VitD3 intake varies by world region, while the prevalence of low serum 25(OH)D, defined as <25/30 and <50 nmol/L, ranges from 5% to 18% and from 24% to 49%, respectively, depending on location and ultraviolet B radiation availability [10,11]. Epidemiological data derived through a genome-wide association study (GWAS) have shown that genetic factors may influence VitD3 deficiency in humans [12]). This process has been associated with the activity of three resistance genes: *CYP24A1*, silencing mediator of retinoic acid and thyroid hormone receptor (*SMRT*), and *SNAIL*, which via various signaling pathways may cause a reduction in circulatory VitD3 levels or a diminution of its availability in target tissues [13]. Hence, globally, large numbers of people could potentially be at risk for VitD3 deficiency.

Based on current data, there appears to be a link between VitD3 deficiency and a predisposition to the development of autoimmune thyroiditis (AIT), including Hashimoto’s thyroiditis (HT), Graves’ disease (GD), and/or postpartum thyroiditis (PPT) [14]. However, despite studies reporting evidence of this association, others have challenged the above findings [15]. In any case, assuming that such an association does exist, it remains to be confirmed whether it underlies a pathological mechanism, reveals a causal relationship, or is merely a result of autoimmune processes [16].

The primary aim of this review is to examine the contribution of VitD3 to, and possibly causative role in, the development of thyroid autoimmunity, while also exploring the possibility that low VitD3 status may exacerbate inflammation of the thyroid gland and could, additionally, be associated with immunosuppression.

## 2. Methods

A literature search was conducted using the PubMed and Scopus databases for articles with the search terms “vitamin D”, vitamin D receptor”, “polymorphisms”, “Hashimoto’s thyroiditis”, “Graves’ disease”, and “autoimmune thyroiditis”, alone or in combination. The resulting articles were reviewed, taking into consideration the quality and ranking of each journal. Emphasis was placed on research conducted over the past decade. The current study was exempt from Hospital Scientific Board approval, given its review form.

## 3. VitD Biosynthesis and Metabolism

**Figure 2:**
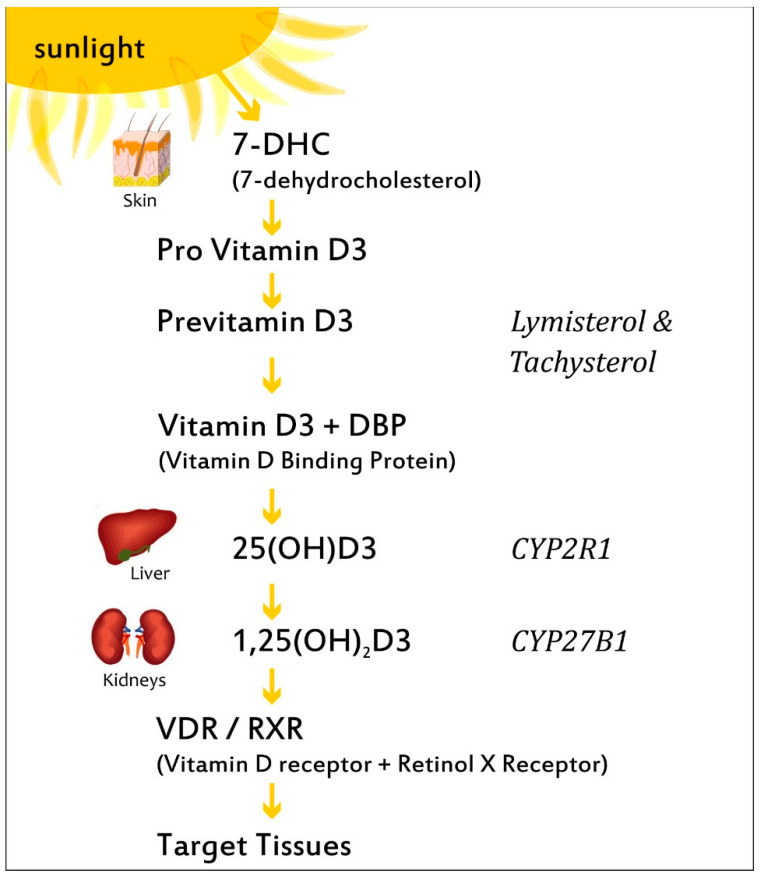
The biosynthesis and metabolism of VitD. Ultraviolet radiation stimulates provitamin D3 to previtamin D3 in the epidermis, which can be either photolyzed to lymisterol and tachysterol or isomerized to VitD3. VitD3 is transported by a binding protein (DBP) to the liver, where it is hydroxylated by CYP2R1 to calcidiol (25(OH)D3). Subsequently, 25(OH)D3 is transported by the DBP to the kidney, where it is hydroxylated in the proximal renal tubule by CYP27B1 (1-α-hydroxylase), resulting in calcitriol, 1,25(OH)_2_D3), the hormonally active form.

VitD is found in plants as ergocalciferol (VitD2), while, in the form of cholecalciferol (VitD3), it can be taken through the diet via regular consumption of dairy products, cereals, egg yolk, and fatty fish such as salmon, mackerel, and sardines. During exposure to sunlight, ultraviolet radiation penetrates into the epidermis and photolyzes provitamin D3 to previtamin D3, which can be either photolyzed to lymisterol and tachysterol or isomerized to VitD3 [17]. The synthesis of VitD3 by the skin depends on a number of factors, including time of day, season, latitude, and skin pigmentation [18]. VitD3 is transported by a specific binding protein (DBP) to the liver, where it is hydroxylated by cytochrome P450 to VitD 25 hydroxylases (including the key enzymes CYP2R1, CYP2D11, and CYP2D25) producing calcidiol, also known as 25-hydroxyvitamin D3 (25(OH)D3) (Figure 2) [5]. Calcidiol is the main metabolite, its circulating form being used to evaluate serum VitD3 status. Following this, 25(OH)D3 is transported by the DBP to the kidney, where it is hydroxylated in the proximal renal tubule by the cytochrome P450 monooxygenase 25(OH)D 1-α-hydroxylase (CYP27B1; 1α(OH)ase), resulting in calcitriol, the hormonally active form, and 1,25-dihydroxyvitamin D3, (1,25(OH)_2_D3), which is responsible for the pleiotropic effects of VitD3 in many systems and organs. Calcitriol binds to the VitD3 receptor (VDR), which subsequently forms a heterodimer with the retinoid-X receptor, and the complex then enters the nucleus and binds to VitD3-response elements (VDREs) in genomic DNA [19].

Although 1-α-(OH)ase is present predominantly in the kidneys, it is also located in extrarenal sites, such as the placenta, leucocytes, macrophages generating 1,25(OH)_2_D3, which may exert immunomodulating action on various immune cells, and particularly agranulocytes such as monocytes [20]. Of note, it is hypothesized that a more complete understanding of VitD3 metabolism and hydroxylase’s function is crucial to explaining the mechanisms of action of 1,25(OH)_2_D3 in various tissues and the immune system.

## 4. VitD3 and Autoimmune Thyroiditis

### 4.1. VitD3 and the Epidemiology of Autoimmune Thyroiditis

One of the first studies in humans was carried out by Kivity S et al. in 2011: the authors observed that VitD3 deficiency was significantly higher in autoimmune thyroid disease (AITD) patients compared to healthy individuals (72% vs. 30.6%; *p* < 0.001) [21]; they also noted a correlation between deficient VitD3 status, antithyroid peroxidase antibody (TPOAb), and abnormal thyroid function tests (*p* = 0.059). One year later, however, Efraimidis G. et al. conducted (A) a cross-sectional study comparing euthyroid subjects with genetic susceptibility for AITD, though without thyroid antibodies, with controls, while also carrying out (B) a longitudinal study comparing patients who had developed de novo thyroid antibodies with those who had not [22]. In neither group were the early stages of thyroid autoimmunity (in study A genetic susceptibility and in study B development of TPOAb) associated with low VitD3 levels.

VitD3 deficiency has been associated with AITD, particularly in premenopausal women with HT [23,24], with several recent trials providing good evidence of a bidirectional association.

A recent epidemiological survey with 1812 participants conducted in Tianjin, China, showed, by logistic regression analysis, that TPOAb positivity was associated with 25(OH)D3 deficiency (odds ratio (OR): 2.428, 95% confidence interval (CI): 1.383–4.261) and 25(OH)D3 inadequacy (OR: 1.198, 95% CO: 0.828–1.733; *p* = 0.008) [25].

In 2015, two cross-sectional case-control studies by the same group, including 70 patients each with newly diagnosed HT and GD, were carried out, while a nested case–control study was conducted in which the levels of 25(OH)D3 were compared between 610 women who developed PPT during the follow-up after delivery and those who did not [14]. In none of these studies did the serum 25(OH)D3 levels reveal any association with TPOAb or with antithyroglobulin antibody (TgAB) nor was any association found with the subjects’ levels of thyroid hormones or with thyroid-stimulating hormone (TSH) in GD and HT. Importantly, however, it was noted that the lower the VitD3 level is, the higher the risk of developing AITD was. Also of interest was the finding that with each 5 nmol/L increase in serum 25(OH)D3 concentration, a fold reduction was observed, with a 1.55, 1.62, and 1.51 increase in GD, HT, and PPT risk, respectively [14].

In an analysis of six clinical trials including 258 patients with HT, a significant difference was found between the 25(OH)D3 levels in the HT group when compared with those of the control group (95% CI: 12.43, 25.58, *p* < 0.001) [15]. Moreover, the combined results of the analysis indicated that VitD3 supplementation may significantly reduce TPOAb titers. It is, however, noteworthy that no significant association was observed between VitD3 serum levels and those of TgAB, TSH, free triiodothyronine (FT3), and free thyroxine (FT4), suggesting that VitD does not affect thyroid function in patients with HT [15].

In contrast, in another study in which 5230 patients were enrolled, 25(OH)D3 levels were higher in the non-HT group than in the HT group [26]. Multiple regression analysis demonstrated that HT was statistically significantly correlated with male gender, body mass index (BMI), waist circumference, TSH, and FT3 and FT4 levels in the insufficiency group and deficiency group. An increase of 25(OH)D3 by 1 ng/mL at the normal reference level was reported to correspond to an increase of 2.78 ng/dl in FT4 concentration and a decrease of 0.17 mIU/L in TSH [26]. Meanwhile, TSH was negatively correlated with 25(OH)D3 concentrations, while FT3 and FT4 levels were observed to be positively correlated with 25(OH)D3 levels.

A possible association between low VitD3 levels and HT was studied in 261 healthy overweight and obese subjects (200 women and 61 men) [27]. VitD3 deficiency was found in 55% of all subjects (144/261), 17% of whom (45/261) had HT. The percentage of subjects with VitD3 deficiency was significantly higher among those with HT (31/45, 69%) compared to those without HT (113/216, 52%) (*p* = 0.042). The study results strongly pointed to VitD3 deficiency being significantly related to HT in overweight and obese individuals, thus confirming previous findings that obesity is associated with lower VitD3 circulating levels [27]. Hence, obese patients with VitD3 deficiency should be checked for the possible presence of HT.

In line with the latter observations, it was demonstrated that long-standing HT patients on levothyroxine (LT4) treatment had far lower 25(OH)D3 levels (11.4 ± 5.2 ng/mL) compared to newly diagnosed HT subjects (13.1 ± 5.9 ng/mL, *p* = 0.002) and control subjects (15.4 ± 6.8 ng/mL, *p* < 0.001) [28]. Serum 25(OH)D3 levels were inversely correlated with TgAB levels (r = −0.335, *p* < 0.001). The severity of the 25(OH)D3 deficient state correlated with the duration of HT, thyroid volume, and antibody levels, indicating a potential role of VitD3 in the development of HT and/or its progression to hypothyroidism.

In the Survey on Prevalence in East China for Metabolic Diseases and Risk Factors (SPECT-China) cross-sectional study, which was performed in 23 sites in East China and included 10,636 participants, four 25(OH)D3-related and four TPOAb-associated single nucleotide polymorphisms (SNPs) were genotyped, and their genetic risk scores were estimated (GRS) [29]. Bidirectional Mendelian randomization (MR) analysis was performed, which showed a significant association of GRS with 25(OH)D3 (B −0.093, 95% CI −0.111 to −0.074) and TPOAb level (B 0.067, 95% CI 0.002 to 0.132). TPOAb GRS was significantly associated with TPOAb concentration (B 0.345, 95% CI 0.135 to 0.556), but not with 25(OH)D (B −0.030, 95% CI −0.091 to 0.030). By applying 25(OH)D3 GRS in the MR analysis, a causal relationship between genetically determined 25(OH)D3 and increased TPOAb concentration was detected. A higher VitD3 GRS was associated with a higher risk of TPOAb positivity, supporting a causal association between decreased VitD3 and increased concentration of TPOAb in the study population [29]. These results provide some evidence that VitD3 supplementation may lessen AITDs susceptibility; if the findings are confirmed, they will be of considerable benefit to public health, considering the wide prevalence of VitD3 deficiency.

VitD3 levels, as well as rates of VitD3 deficiency, were compared between 461 HT cases and 176 controls drawn from a Croatian Biobank of HT patients (CROHT) [30]. HT patients were additionally divided into two groups, mild and overt, in order to take into account HT severity. Although no significant differences in VitD3 levels or rates of VitD3 deficiency were detected between HT patients and controls, a clearly significant difference was observed between mild and overt subgroups for VitD3 levels (OR = 1.038, *p* = 0.023). These data may suggest that prolonging VitD3 deficiency may advance hypothyroidism.

Concerning GD, an experimental study conducted by Misharian et al. [31] investigating the relationship between VitD and autoimmunity made an unexpected finding: VitD3-deficient BALB/c (albino, laboratory-bred strain) mice developed persistent hyperthyroidism following immunization with the TSH receptor, while the sufficient VitD3 group did not. It was these unanticipated results that led to the hypothesis that low VitD3 levels may contribute to the development of AITD.

A cross-sectional study was carried out in 2013, recruiting 54 patients with GD, of whom 18 were in remission (R) and 36 in non-remission (NR), and 49 controls (C) [32]. The authors reported that serum 25(OH)D3 levels were significantly lower in the NR group than in the R and C groups (14.5 ± 2.9 vs. 18.2 ± 5.1 ng/mL, *p* < 0.005, and 18.6 ± 5.3 ng/mL, *p* < 0.0005, respectively) [32]. However, they found no significant association between serum 25(OH)D3 levels and TSH receptor antibodies (TRAb) levels in NR. Based on the above results, any association of VitD3 with disease severity appears to be unlikely.

In 2018, a study compared VitD3 levels in 292 patients with newly diagnosed GD and in 2305 controls, while in 708 patients and 1178 controls, SNPs in the *VDR*, *DBP*, and *CYP27B1 recepto*r genes were examined to determine whether there was any association with GD and/or Graves’ ophthalmopathy (GO) [33]. The investigators observed, by performing a genetic analysis, that two single SNPs in *VDR*, namely that *rs10735810* (OR = 1.36, 95% CI: 1.02–1.36, *p* = 0.02) and *rs1544410* (OR = 1.47, 95% CI: 1.03–1.47, *p* = 0.02) were indeed associated with GD. Meanwhile, no difference was observed in the mean VitD3 levels between genotypes in either *rs10735810* or *rs154410* carriers.

The existing data appears to point to a higher prevalence of VitD3 deficiency in patients with GD. However, the role of VitD3 in GD is still an unresolved issue, as the association does not necessarily imply a causal relationship [34].

### 4.2. Effects of VitD3 Treatment on Hashimoto’s Thyroiditis and Graves’ Disease

A case-control study enrolled 218 subjects and supplemented them with cholecalciferol over a period of 4 months. [35]. The results revealed, firstly, that there was a significant negative correlation only between serum 25(OH)D3 levels and TPOAb and, secondly, that TPOAb levels were significantly higher in 186 of the subjects who had HT together with VitD3 deficiency compared to 32 HT patients with no VitD3 deficiency (364 ± 181 IU/mL vs. 115.8 ± 37.1 IU/mL, *p* < 0.0001). Cholecalciferol supplementation in the 186 VitD3-deficient patients led to a significant decrease, by 20.3%, in serum TPOAb titers. At the conclusion of the 4-month study period, BMI, serum TGAB, and TSH levels had decreased by 2.2%, 5.3%, and 4%, respectively, though no significance was achieved.

A double-blind, randomized, placebo-controlled clinical trial enrolling 56 VitD3-deficient euthyroid or hypothyroid patients with positive TPOAb studied the effects of VitD3 treatment over a 12-week period [36]. The subjects were randomly allocated to two groups, numbering 33 and 32 participants, which received oral VitD3 (50,000 IU weekly) (“VitD-treated group”) and a placebo (“placebo group”), respectively. The mean (standard error) of VitD increased significantly in the VitD3-treated group (45.53 (1.84) ng/mL vs. 12.76 (0.74) ng/mL, *p* = 0.001). No improvement was noted in any metabolic parameter following 12 weeks of high-dose VitD3 supplementation in the HT patients with a VitD3 deficiency [36].

In 2019, a study was carried out to ascertain whether VitD3 supplementation was able to modify the circulating thyroid autoantibodies and thyroid profile in female patients with HT [37]. Forty-two women with HT were enrolled and randomly assigned to two groups: a VitD3 group receiving 50,000 IU VitD3 and a placebo group receiving placebo pearls, taken weekly for a period of 3 months [37]. Although a significant decrease in TgAb and TSH was observed in the VitD3-treated group, there was no noteworthy reduction of TPOAb in this group compared to the placebo group nor was there any significant change in the thyroid hormone concentrations in the VitD3-treated group.

It may, thus, be concluded that short-term high-dose VitD3 supplementation may be capable of reducing HT activity, albeit further confirmation of the latter results should be obtained via large randomized controlled trials.

In GD, a double-blinded clinical trial was set out to investigate the effects of VitD3 supplementation on the muscle weakness and quality of life (QoL) impairments that are frequently observed in GD patients [38]. Patients were randomized to VitD3 70 μg (2800 IU)/day or a matching placebo as an add-on to standard antithyroid drug (ATD) treatment at baseline and during a period of 3 and 9 months of follow-up. Whereas VitD3 supplementation in fact decreased muscle strength and did not improve QoL, the ATD treatment normalized muscle function and improved lean body mass [38].

In an analysis to study the association between VitD3 deficiency and HT and GD, 11 case-control studies that included 1952 AITD patients with HT or GD were reviewed [39]. The majority of the studies analyzed revealed that HT and GD patients have a greater prevalence of VitD3 deficiency or low serum 25(OH)D3 levels. Other studies, however, failed to establish an association between VitD3 deficiency and HT and GD [39].

In patients with GD, it is advisable to routinely measure serum VitD3 concentrations, particularly in smokers and in those with increased titers of thyrotropin receptor autoantibodies (TRAb) and/or with GO. If VitD3 deficiency is registered, low-dose VitD3 supplementation should be administered [40]. While VitD3 has a remarkable ability to modulate both innate and adaptive immune responses, since VitD3 deficiency is associated with increased autoimmunity incidence and susceptibility to infection, it must be supplemented cautiously. Given that taking high VitD3 supplementation over a long period of time can induce hypercalcemia, a moderate dose, i.e., 10 micrograms a day, is advised for most people [41,42], and monitoring of calcium levels, particularly in older patients, is recommended [42].

### 4.3. VitD3 and Pregnancy

VitD3 deficiency in pregnancy has been associated with pre-eclampsia, preterm delivery, gestational diabetes mellitus, and small-for-gestational-age births and is a risk factor for AITD, although a causative relationship in the development of AIT has not to date been clearly demonstrated [43]. The modulation of immune responses by 1,25(OH)_2_D3 consolidates T regulatory cells (Tregs) function, while inhibiting inflammatory responses of Th17 cells, which may reduce the risk of unexplained recurrent spontaneous abortion.

In a retrospective cross-sectional study of 133 women with repeated spontaneous abortions before 20 weeks, gestation serum VitD3 levels were measured, and autoimmune parameters were monitored [44]. It was observed that 63 of the women (47.4%) had low VitD (<30 ng/mL) and that the prevalence of antiphospholipid antibody (APA) in the low VitD group (VDlow) was significantly higher (39.7%) than in the group with normal VitD levels (VDnl) (22.9%) (*p* < 0.05). It was also noted that prevalence of antinuclear antigen antibody (VDlow vs. VDnl; 23.8 vs. 10.0%, OR 2.81, 95% CI 1.1–7.4), anti-ssDNA (19.0 vs. 5.7%, OR 3.76, 95% CI 1.1–12.4), and TPOAb (33.3 vs. 15.7%, OR 2.68, 95% CI 1.2–6.1) was significantly higher in the VDlow group than in the VDnl group. However, no difference in Th1/Th2 ratios between the VDlow and VDnl groups was found [44].

In another study, serum samples of 50 women were selected retrospectively, and VitD3 levels were measured at gestational weeks 8, 20, and 32 [45]. The median 25(OH)D3 levels were lower in the first trimester (28.29 nmol/L) than in the second (39.23 nmol/L) or third (40.03) trimester. Only triiodothyronine was associated with VitD3 in the first trimester (*p* = 0.024), with only a statistically significant trend detected (*p* = 0.063). No association was observed between VitD3 and any other thyroid parameters [45]. Although large studies are needed, the data pointed to adequate dietary supplementation during the entire period of pregnancy, particularly for those with autoimmune diseases and women in the first trimester.

## 5. VitD3 and Mechanisms of Autoimmunity

VitD3 has a significant positive impact on immunity, with moderate dietary intake being proven to increase both innate and adaptive immune responses [46]. Recent evidence demonstrated that VitD3 seems to exert an immunomodulating action on autoimmune diseases and cancers [47].

**Figure 3:**
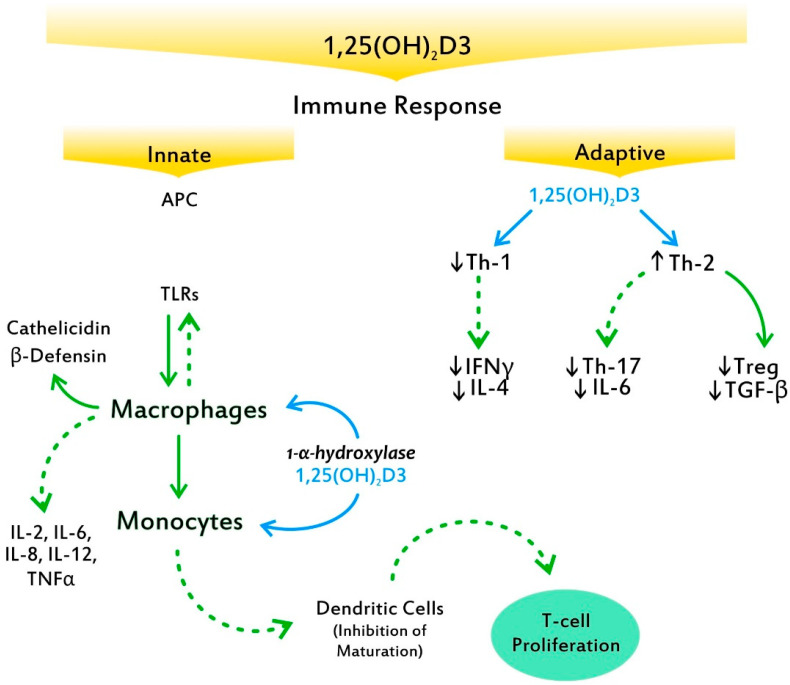
Toll-like receptors (TLRs) stimulated by antigens begin a cascade that results in the production by macrophages of peptides with potent bacterial activity such as cathelicidin and β-defensin. 1,25 (OH)_2_D3 generated by 1-α-hydroxylase in the monocyte and macrophage suppresses T cell proliferation via the induction of tolerogenic dendritic cells, resulting in a shift from a Type 1 T helper (Th1) phenotype to a Type 2 T helper (Th2) phenotype. Moreover, it affects T cell maturation with a skewing away from the inflammatory Th1-7 cytokine, while it facilitates the induction of anti-inflammatory T regulatory cells (Tregs). Solid arrows indicate a positive effect. Dotted arrows indicate an inhibitory effect.

VitD3 plays a central role in the innate antimicrobial response. 1,25(OH)_2_D3 is able to redirect the differentiation of monocytes away from dendritic cells (DCs) and toward macrophages, which elevate antimicrobial activity by increasing the production of antimicrobial peptides, such as cathelicidin and β-defensin 4 (Figure 3) [48]. The recently discovered ability of VitD3 to promote cathelicidin synthesis, a pivotal finding in evolutionary development, is crucial, as this protein possesses several functions, including stimulation of the chemotaxis of neutrophils, monocytes, macrophages, and T cells at the site of infection [49]. Indeed, the enzyme CYP27B1 is expressed in the airway epithelium and alveolar macrophages and produces both 1,25(OH)_2_D and VDR, which subsequently stimulate the innate immune response, providing the first line of defense against viral and bacterial infections [50]. Through additionally downregulating antigen presentation by monocytes, calcitriol enhances immune tolerance [51]. Moreover, it upregulates intracellular calcium and nitric oxide, stimulates phosphatidylinositol 3-kinase catalytic subunit type 3 (PI3KC3) activity, and induces chemotaxis and autophagy of innate immune cells, thereby promoting the clearance of pathogens [52]. The above compose key factors behind VitD3′s ability to modulate numerous immune mechanisms, thereby containing the virus. Notably, VitD3 regulates the renin–angiotensin system, which is exploited by severe acute respiratory syndrome coronavirus-2 (SARS-CoV-2) for entry into the host cells [53].

In the adaptive immune response, VitD3′s ability to suppress T cell proliferation results in a shift from a Th1 phenotype to a Th2 phenotype [54]. Furthermore, it affects T cell maturation with a skewing away from the inflammatory Th17 phenotype, while it facilitates the induction of Tregs [55]. These effects result in decreased production of inflammatory cytokines (IL-17, IL-21) (Figure 3). VitD3 also has effects on monocytes and DCs, inhibiting monocyte production of inflammatory cytokines, such as IL-2, IL-6, IL-8, IL-12, and tumor necrosis factor alpha (TNFα), as well as inhibiting the maturation of DCs [56]. Among the major effects exerted by 1,25(OH)_2_D3 on B cells is calcitriol’s ability to induce B cell apoptosis by arresting the proliferation and differentiation of cytokines into plasma cells [57]. Furthermore, their suppression of DCs’ differentiation and maturation is fundamental to autoimmunity as well as to the abrogation of self-tolerance. Interestingly, while antigen presentation to a T cell by mature DCs enables the triggering of an immune response against the same antigen, antigen presentation by immature DCs promotes tolerance [58]. Of note, presentation of these self-antigens is generally by immature DCs; thereby, tolerance to self is maintained.

DCs are specialized sentinel cells that play a central role in shaping the adaptive immune response, by being critical for the regulation of T cell activation. Both mouse and human DCs generated in the presence of 1,25(OH)_2_D3 upregulate the cluster of differentiation 31 (CD31) expression, which leads to the reduced ability to prime CD4+ T cells by impairing cell–cell contact [59].

Moreover, 1,25(OH)_2_D3 showed an anti-proliferative effect in T-helper cells that was blocked by sirtuin 1 histon deacethylase (SIRT1) inhibition (T-helper cells: *p* = 0.0059), with simultaneously elevation of *transcription factor forkhead box class O3a (FOXO3a*) gene expression in T-helper cells. SIRT1 is apparently the link between VitD3 and *FOXO3a* regulation, suggesting an important role of the SIRT1-FOXO3a axis in the protective activity of VitD3 in autoimmunity [60].

Recently, the results of the first large-scale, systematic investigation of genetic determinants of VitD3 deficiency were reported [61]. The analysis of the genetic architecture of this trait may enhance our understanding of the regulation of VitD3 metabolism. Variants near the genes involved in cholesterol synthesis, hydroxylation, and VitD3 status are potentially capable of identifying individuals with a substantially higher risk of VitD3 deficiency and probably AITD.

In another analysis, GWAS summary datasets for serum lipids were obtained from the Global Lipids Genetics Consortium (GLGC), and VitD3 deficiency data were acquired from the UK Biobank samples [62]. Single-variable MR (SVMR) and multi-variable MR (MVMR) analyses were performed, revealing that high serum triacylglycerol (inverse variance weighted (IVW), OR = 0.85, 95% CI: 0.81–0.89, *p* < 0.001), low-density lipoprotein (LDL) (IVW, OR = 0.93, 95% CI: 0.90–0.95, *p* < 0.001), and high-density lipoprotein (HDL) (IVW, OR = 0.95, 95% CI: 0.91–0.98, *p* < 0.001) levels had a causal relationship with VitD3 deficiency [62]. Although there are some limitations, further investigations on this topic are noteworthy.

As VDR gene polymorphisms have been associated with AITD, any links between VitD deficiency and AITD might be due either to gene polymorphisms or to environmental factors, such as lack of dietary intake and sun exposure.

What is more, in the aging process as well as in renal diseases, the kidneys gradually lose their capacity to convert 25(OH)D3 to 1,25(OH)_2_D3; this leads to increased renal metabolism of 1,25(OH)_2_D3 [63]. In the elderly, this mechanism may contribute to a VitD3 deficient state, which renders older people, particularly those with susceptibility, more prone to infection, frailty, and autoimmune diseases.

## 6. Discrepancies and Limitations

There are, thus far, no scientific data demonstrating a clear benefit of VitD3 supplementation in AIT, although ample evidence exists showing that states of VitD3 deficiency may disrupt both the innate and adaptive responses. The increased mortality reported among patients with very low VitD3 and virus diseases and among women with adverse pregnancy outcomes constitutes adequate confirmation of this. Animal studies have reported that VitD3 supplementation may delay progression of AIT; on the other hand, observational studies in humans did not show any significant effect [64,65,66]. These discrepancies may be due to the considerable heterogeneity of the studies regarding size, gender, degree of disease, BMI, dose, and duration of treatment. The onset of AIT might also be of importance, as longstanding and advanced AIT may be refractory to VitD3 supplementation.

Recently, the VITamin D and OmegA-3 TriaL (VITAL), a nationwide, randomized, blind, placebo-controlled trial with 25.871 participants revealed that VitD3 supplementation, with or without omega 3 fatty acids, taken over five years, reduced autoimmune disease (AID) by 22%, while omega 3 fatty acid intake decreased AIT by 15% (statistically non-significant) [67]. Of note, both treatment arms showed larger effects than the reference arms (VitD3 placebo and omega 3 fatty acid placebo). Taking into account that only the last years of the intervention were considered, the VitD3 group had 39% fewer participants with confirmed AID than the placebo group (*p* = 0.005), while the omega 3 fatty acid group had 10% fewer participants with confirmed AID than the placebo group [67]. These results may suggest a synergistic effect of VitD3 with other nutrients in modulating the autoimmune process. 1,25(OH)_2_D3, generated by 1-α-hydroxylase in various immune cells, acts as an endocrine hormone by serving as a high-affinity ligand to the transcription factor VDR. Binding to VDR, 1,25-dihydroxyvitamin D regulates an array of genes, many of which are involved in inflammation and acquired and innate immune responses. Thus, 1,25(OH)_2_D3 can potently inhibit pathogenic T cells and greatly increases the numbers of Tregs [68]. Any statistically significant effect can take some time, though it may be accelerated by other nutrients, such as selenium and or/zinc, that possess antioxidant and anti-inflammatory properties. It, however, remains uncertain whether supplementation with VitD3 prevents autoimmunity, cancer, or cardiovascular disease, due to the fact that large-scale randomized trials of VitD3 at moderate or high doses are lacking. The VITAL study was conducted at a dose of VitD3 2.000 IU per day, with omega-3 fatty acids at a dose of 1 g per day, for the prevention of cancer and cardiovascular disease among men and women aged 50 and 55 years and older, respectively [69]. Primary end points were invasive cancer of any type and major cardiovascular events; secondary end points included death from cancer and additional cardiovascular events. The results did not reveal any effect on the incidence of advanced cancer or cardiovascular events.

## 7. Conclusions

The majority of recent research findings have revealed an association between VitD3 levels and the immunological parameters of AITD, which, however, does not necessarily imply causality. VitD3 levels were seen to be lower in longstanding HT patients, while in the early stages of the disease, no direct association was found. Importantly, though patients with GD have lower VitD3 levels compared to the general population, the concentrations do not affect the laboratory or clinical parameters of GD or GO. SNPs in the VDR are observed to influence the risk of GD through mechanisms other than by reducing VitD3 levels. Both GD with thyrotoxicosis and HT with hypothyroidism exhibited decreased 25(OH)D levels, suggesting that, in the context of an individualized approach, patients with AITD should be checked for VitD3 deficiency; if confirmed, it should be supplemented, preferably via their diet, or with Vitd3 compounds at a moderate dose, i.e., 1.000–2.000 U/day, and be periodically monitored over a long period. Overall, small-to-moderate amounts of VitD3 are considered adequate and safe, and, among the healthy population, most people do not need screening or supplements.

The links between VitD3, inflammation, and AIT and the implications involved are topics that continue to arouse interest among researchers worldwide. However, before new large trials are organized, ongoing controversies in VitD3 research, such as the homogeneity of the assays applied to determine serum 25(OH)D3 concentration and the definitions of VitD3 nutritional status, i.e., insufficiency, deficiency, or excess, should be addressed based on the revisions of the last Consensus Statement from the 2nd International Conference on Controversies in VitD3 [70].

## Data Availability

Not applicable.

## Abbreviations

AID	autoimmune disease
AIT	autoimmune thyroiditis
AITD	autoimmune thyroid disease
DBD	vitamin D binding protein
CYP27B1	(1-α-hydroxylase)
GWAS	genome-wide association studies
GD	Graves’ disease
GO	Graves’ opthalmopathy
HT	Hashimoto’s thyroiditis
IL-6	interleukin 6
SNP	single-nucleotide polymorphism
TgAB	antithyroglobulin antibodies
TNFα	tumor necrotic factor α
TPOAb	antithyroid peroxidase antibodies
TRAb	TSH receptor antibodies
Tregs	T regulatory cells
TSH	thyroid-stimulating hormone
VDR	vitamin D receptor
